# Could albumin level explain the higher mortality in hemodialysis patients with pulmonary hypertension?

**DOI:** 10.1186/1471-2369-13-80

**Published:** 2012-08-06

**Authors:** Hugo Hyung Bok Yoo, Luis Cuadrado Martin, Ana Claudia Kochi, Lidiane Silva Rodrigues-Telini, Pasqual Barretti, Jacqueline Teixeira Caramori, Beatriz Bojikian Matsubara, Silméia Garcia Zannati-Bazan, Roberto Jorge da Silva Franco, Thais Thomaz Queluz

**Affiliations:** 1 Division of Pulmonology, State University of São Paulo - UNESP, Botucatu School of Medicine, Botucatu, SP, Brazil; 2 Division of Nephrology, State University of São Paulo - UNESP, Botucatu School of Medicine, Botucatu, SP, Brazil; 3 Division of Cardiology, State University of São Paulo - UNESP, Botucatu School of Medicine, Botucatu, SP, Brazil

**Keywords:** End-stage renal disease, Hemodialysis, Pulmonary hypertension, Prognostic

## Abstract

**Background:**

The pathogenesis of pulmonary hypertension (PH) in hemodialysis is still unclear. The aim of this study was to identify the risk factors associated with the presence of PH in chronic hemodialysis patients and to verify whether these factors might explain the highest mortality among them.

**Methods:**

We conducted a retrospective study of hemodialysis patients who started treatment from August 2001 to October 2007 and were followed up until April 2011 in a Brazilian referral medical school. According to the results of echocardiography examination, patients were allocated in two groups: those with PH and those without PH. Clinical parameters, site and type of vascular access, bioimpedance, and laboratorial findings were compared between the groups and a logistic regression model was elaborated. Actuarial survival curves were constructed and hazard risk to death was evaluated by Cox regression analysis.

**Results:**

PH > 35 mmHg was found in 23 (30.6%) of the 75 patients studied. The groups differed in extracellular water, ventricular thickness, left atrium diameter, and ventricular filling. In a univariate analysis, extracellular water was associated with PH (relative risk = 1.194; 95% CI of 1.006 – 1.416; p = 0.042); nevertheless, in a multiple model, only left atrium enlargement was independently associated with PH (relative risk =1.172; 95% CI of 1.010 – 1.359; p = 0.036). PH (hazard risk = 3.008; 95% CI of 1.285 – 7.043; p = 0.011) and age (hazard risk of 1.034 per year of age; 95% CI of 1.000 – 7.068; p = 0.047) were significantly associated with mortality in a multiple Cox regression analysis. However, when albumin was taken in account the only statistically significant association was between albumin level and mortality (hazard risk = 0.342 per g/dL; 95% CI of 0.119 – 0.984; p = 0.047) while the presence of PH lost its statistical significance (p = 0.184). Mortality was higher in patients with PH (47.8% vs 25%) who also had a statistically worse survival after the sixth year of follow up.

**Conclusions:**

PH in hemodialysis patients is associated with parameters of volume overload that sheds light on its pathophysiology. Mortality is higher in hemodialysis patients with PH and the low albumin level can explain this association.

## Background

Pulmonary hypertension (PH) comprises a group of clinical and pathophysiological entities with similar features due to a great variety of underlying conditions [1,2]. Chronic renal disease is one of these causes [3] but the pathogenesis of PH in this group of patients is still unclear. Hemodialysis patients are exposed to continuous pulmonary insults of multifactorial origin, such as hormonal and metabolic derangement associated with end-stage terminal disease, which may induce pulmonary vascular alterations and consequent increased resistance. Besides, high serum level of acute phase reactive protein and cytokines, including IL-1β, TNF-α and IL-6 have been demonstrated in this population suggesting that chronic inflammation might have some role in the pathogenesis of PH in patients undergoing hemodialysis [4]. On the other side, PH can cause right ventricular failure with clinical manifestations of systemic venous congestion, pleural effusion, and ascites [1] which might also result in reduced systemic arterial pressure and intradialytic hypotension [2,5]. In fact, renal transplantation, by restoring normal renal function, is one of the most effective choices of treatment for PH in end-stage renal disease patients [6].

PH has been associated with poor survival in hemodialysis patients [3,6-9]. However, data regarding the prevalence and the influence of others factors that can affect the survival of PH patients with end-stage renal disease are limited. Hence, the aim of this study is to identify the risk factors associated with the presence of PH in hemodialysis patients and to verify whether these factors might explain the highest mortality among these patients.

## Methods

We retrospectively collected demographic, clinical, and laboratorial data from the charts of hemodialysis patients of the Division of Nephrology of the University Hospital of the Botucatu School of Medicine, State University of São Paulo, Brazil. Patients had started treatment from August 2001 to October 2007 and were followed up until April 2011. All patients had at least one monthly appointment with their nephrologists at the hospital’s clinics.

The inclusion criteria were patients older than 18 years, in the hemodialysis program for over two months, who had a good technical quality echocardiography examination. The exclusion criteria were the presence of ventricular dyskinesias and/or hemodynamically significant valvar disease, alcoholics, psychiatric patients, carriers of hepatic cirrhosis and/or malignant neoplasms.

The Research Ethical Committee of the Botucatu Medical School approved the study and granted a waiver for informed consent (n^o^ 3374/09), since it was only a survey of charts.

Demographic and clinical data (comorbidities, type and location of vascular access and bioimpedance) were recorded. Results of blood tests for hematocrit, C-reactive protein, creatinine, albumin, calcium, phosphorus, and parathyroid hormone levels were also collected. All the results recorded were from exams performed at the beginning of the midweek hemodialysis within at least two weeks before or after the echocardiography study.

Based on this examination, patients were allocated in two groups: PH group, formed by patients echocardiographycally diagnosed as having PH, and non PH group, formed by patients with no PH. Clinical and laboratorial findings between these two groups were compared.

Echocardiographic imaging was performed according to a previously standardized technique [10,11] by highly skilled echocardiographers using a Sonos 2000 (Hewlett Packard) equipment attached to a multifrequential 2.5- to 3.5-MHz transducer. The coefficient of variation of echocardiographic measurements in our laboratory was 2.5 %. The following data were registered: diameters of the left ventricular (LV) cavity at systole and diastole; thickness of the posterior wall and the septum, both at diastole and systole, left atrium and aorta at systole; systolic volume; early peak of mitral flow velocity (E wave); atrial peak filling velocity (A wave). These data were used to calculate the relative thickness of the left ventricle, the left atrium/aorta diameter ratio, the E/A relationship, the LV mass, and LV mass index (LVMI). In case of absence of tricuspid regurgitation, a relationship between time with peek flow/time of right ventricular ejection inferior to 0.3 was defined as PH [12]. When tricuspid regurgitation was identified with continuous wave Doppler, systolic pulmonary arterial pressure was calculated using a validated equation: SPAP = 4 X (tricuspid systolic jet)^2^ + 10 mmHg (estimated right atrial pressure) [10]. PH was defined as a systolic pulmonary arterial pressure equal or higher than 35 mmHg [10,11].

Monofrequencial electric bioimpedance (800 μA and 50 kHz) was performed with Biodynamics 450 device (Biodynamics®, USA) after the end of hemodialysis, with the patients in the supine position. Patient’s assessments were conducted using a connection between the analyzer to the back of the hand and instep of the subject. Resistance and reactance were measured; phase angle, total body water (TBW), intra (ICW) and extracellular water (ECW) were calculated based on resistance and reactance. Then a microprocessor uses the stored values to perform subsequent calculations according to the following equations:

(1) $$\text{Phase angle}=\text{arc tangent}\;\left({\text{tan}}^{-1}\right)\;\text{of reactance/resistance}[13];$$

(2) $$\text{Total body water}=\left({\text{height}}^{2}/\text{resistance}\right)+\text{b}\left(\text{weight}\right)+\text{c}\left(\text{age}\right)+\text{d}[14];$$

(3) $$\text{Intracellular water}=\text{a}\;\left({\text{height}}^{2}\right)\;\left(\text{reactance}/{\text{resistance}}^{2}\right)+\text{b}\;\left(\text{weight}\right)+\text{c}\;\left(\text{age}\right)+\text{d}[15];$$

(4) Extracellular water=total body water−intracellular water[15];

(5) $$\text{Lean body mass}=\text{a}\;\left({\text{height}}^{2}\right)+\text{b}\;\left(\text{weight}\right)+\text{c}\;\left(\text{age}\right)+\text{d}\;\left(\text{resistance}\right)+\text{e}\left[13\right]\text{;}$$

(6) Fat mass=weight−fat−free mass[13];

Variables a, b, c, d, and e represent constant coefficients calculated by regression analysis in each instance according to references [13-15].

### Data analysis

Normally distributed variables were described as mean and standard deviation and the frequencies as percentage. Variables not normally distributed were described as median and interquartile interval.

The clinical characteristics evaluated at the beginning and at the end of follow-up within groups were compared by paired t-test; the comparisons between PH group and non-PH group were performed by t-test for unpaired samples, qui-square, or Mann–Whitney test, as appropriate.

Variables with a >10% difference between the two groups were selected for multivariate Cox proportional risk regression analysis. Categorical variables were coded as presence (1) or absence (0) to be included in the Cox model. Potential collinearity among variables selected for multiple analysis were tested and if associations were present one of the variables was excluded from the Cox model. Results were considered significant at P < 0.05. Three Cox models were elaborated: model 1, presence of PH; model 2, presence of PH, age, sex, body mass index, presence of smoking, diabetes and/or dyslipidemia, and systolic blood pressure; model 3, the covariates in model 2 and extracellular water. Primary end point was death by any cause. Patients followed until April 2011 who did not reach the end point were censored. Actuarial survival curves were constructed according to the life table method and compared by Greenwod method [16]. A logistic regression model was constructed to evaluate associations between presence of PH and its risk factors.

Statistical analysis was performed using the Statistical Package for Social Sciences (SPSS) version 12.0 (SPSS, Chicago, IL).

## Results

There were 118 hemodialysis patients eligible for this study, but 43 were excluded for meeting any exclusion criterion. Table 1 shows the clinical characteristics of the 75 patients, 47 males and 28 females, included in the study. Their mean age was 56 ± 12.7 years, there were 53 whites, 16 of them in PH group, 20 afro-descendents, seven in PH group, and two Asiatics, both in non-PH group. There were 18 diabetics, six of them in PH group, 51 dyslipidemics, 15 of them in PH group, and 12 smokers, four of them in PH group. The two groups were not statistically significant different regarding these attributes.

**Table 1:** Clinical and bioimpedance data in groups of hemodialysis patients with and without pulmonary hypertension

	**Pulmonary Hypertension (n = 23)**	**No Pulmonary Hypertension (n = 52)**	**p**
Age (years)	59 ± 11.2	55 ± 13.3	0.189
Male/Female	16/	29/	0.385
White/non White	9/14	18/34	0.909
BMI (Kg/m2)	24.9 ± 3.86	24.4 ± 5.08	0.695
Vascular access			0.777
Radial autologous	18	34	
Brachial autologous	1	5	
Radial graft	1	5	
Femoral graft	2	4	
Catheter	1	4	
IWG (kg)	2.2 (2.0-3.2)	2.8 (2.2-3.4)	0.229
SBP (mm Hg)	142 ± 17.2	147 ± 14.0	0.221
DBP (mm Hg)	86 ± 9.16	89 ± 8.35	0.131
Phase angle (degree)	6.3 ± 1.2	6.6 ± 1.2	0.243
Capacitance	619.0 ± 194.8	635.2 ± 149.3	0.701
Resistance	582.3 ± 104.1	591.8 ± 87.9	0.691
Reactance	62.8 ± 10.7	69.6 ± 16.7	0.087
Cellular mass (kg)	22.2 ± 5.9	20.5 ± 4.7	0.197
Extracellular mass (kg)	25.8 ± 4.9	22.8 ± 4.9	0.019
LM (kg)	48 ± 10.3	43.3 ± 9.0	0.055
FM (kg)	20.7 ± 8.5	20.0 ± 8.7	0.737
ICW (l)	19.1 ± 5.0	17.6 ± 3.7	0.175
ECW (l)	15.7 ± 2.6	14.0 ± 3.2	0.032
TBW (l)	0.857 ± 0.184	0.811 ± 0.176	0.317
ECW/TBW (%)	34.8 ± 6.9	31.668 ± 6.0	0.055

*BMI*: body mass index; *IWG*: interdialytic weight gain; *SBP*: systolic blood pressure; *DBP*: diastolic blood pressure; *LM*: lean body mass; *FM*: fat mass; *ICW*: intracellular water; *ECW*: extracellular water; *TBW*: total body water.

Table 1 also expresses clinical anthropometric and bioimpedance characteristics of groups. PH group patients presented both more extracellular mass and water. The site and type of vascular access did not differ between groups.

Echocardiographic and laboratorial data are shown in Table 2. The groups differed in ventricular thickness, left atrium diameter, and early systolic peak of ventricular filling.

**Table 2:** Echocardiographic and laboratorial data in groups of hemodialysis patients with and without pulmonary hypertension

	**Pulmonary Hypertension (n=23)**	**No pulmonary Hypertension (n=52)**	**p**
LVIDD (mm)	48.6±5.86	49.0±6.57	0.810
PWT (mm)	12.5±1.72	11.5±1.76	0.016
IVST (mm)	13.0±1.74	11.9±2.26	0.042
LVRWT	0.53±0.109	0.49±0.111	0.087
LVM (g)	309±75.5	277±78.8	0.101
LVMI (g/m^2.7^)	81.7±23.51	78.4±23.10	0.575
LA (mm)	46.2±4.29	41.9±5.68	0.002
AO (mm)	34.1±4.59	32.5±3.47	0.102
LA/AO	1.4±0.21	1.3±0.20	0.140
E cm/s	81.1±28.57	65.7±28.53	0.042
A cm/s	87.3±23.88	81.0±18.71	0.256
E/A	0.79(0.70-0.94)	0.74(0.55-0.94)	0.224
PTH (pg/ml)	241(117-491)	257(128-481)	0.726
Creatinine (mg/dl)	10.7±3.16	10.9±3.07	0.811
Albumin (g/dl)	3.6±0.36	3.8±0.41	0.082
Calcium (mg/dl)	9.1±0.80	9.0±0.72	0.756
Phosphorus (mg/dl)	4.6±1.74	5.3±1.57	0.134
Ca x P	42.5±16.94	47.3±14.33	0.204
CRP (mg/dL)	0.8 (0.20-1.27)	0.5 (0.10-1.20)	0.633
Ht (%)	33.6±5.54	33.2±4.31	0.707

*LVIDD*: left ventricular internal diastolic dimension; *PWT*: posterior wall thickness; *IVST*: interventricular septum thickness; *LVRWT*: left ventricular relative wall thickness; *LVM*: left ventricular mass; *LVMI*: left ventricular mass index; *LA*: left atrium; *AO*: aorta; *E*: early peak diastolic flow; *A*: atrial peak diastolic flow; *PTH*: parathyroid hormone; *Ca x P*: calcium phosphorus product; *CRP*: C-reactive protein; *Ht*: hematocrit.

The associations of PH with left ventricular hypertrophy, diastolic dysfunction, and volume status were assessed by logistic regression. Firstly, in an univariate analysis, ECW was statistically associated with PH (relative risk of 1.194; 95% confidence interval of 1.006 – 1.416; p = 0.042). Secondly, including left atrium this variable was statistically associated with the presence of PH (relative risk of 1.216; 95% confidence interval of 1.057 – 1.398; p = 0.006) and ECW lost its significance (p = 0.303). In subsequent models, in which were successively included diastolic filing of left ventricle, albumin level, and posterior wall thickness only left atrium maintained statistically significant association with PH (relative risk of 1.172; 95% confidence interval of 1.010 – 1.359; p = 0.036).

**Figure 1:**
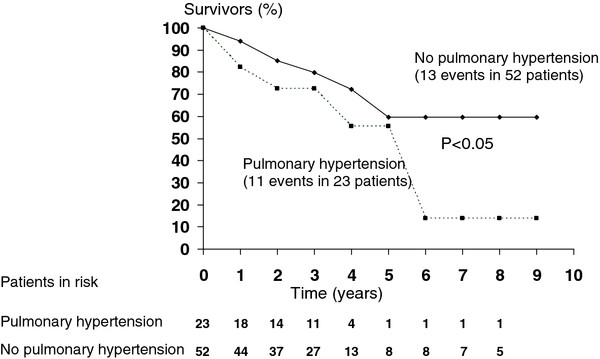
Mortality associated with pulmonary hypertension in hemodialysis patients.

Among the 23 patients with PH there were 11 deaths (47.8%) while among the 52 patients without PH there were 13 deaths (25%). Survival table curves concerning the presence or absence of PH are expressed in Figure 1. PH patients had a statistically worse survival after the sixth year of follow up.

Cox regression analysis, which included variables with p < 0.1 between groups, revealed a statistically significant association only between albumin levels and mortality (Table 3).

**Table 3:** Cox regression: all mortality causes in hemodialysis patients

	**Hazard ratio**	**95.0% CI for HR**		**p-value**
		**Lower**	**Upper**	
Albumin (g/dL)	0.117	0.019	0.714	0.020
Relative wall thickness	0.039	0.000	3.78 x 10^2^	0.488
Posterior wall (mm)	1.515	0.841	2.730	0.167
Interventricular septum (mm)	1.055	0.587	1.898	0.858
Left atrium (mm)	0.925	0.806	1.060	0.261
Early peak diastolic (cm/s)	0.992	0.971	1.013	0.435
PH (presence/absence)	1.734	0.555	5.415	0.344
ECW/TBW	2.1x10^3^	0.001	4.1x 10^9^	0.299
Intracellular water (L)	0.817	0.582	1.146	0.241
Height (m)	0.996	0.918	1.081	0.932
Reactance (Ω)	0.991	0.937	1.048	0.755

*PH*: pulmonary hypertension; *ECW/TBW*: extracellular water/total body water.

The Cox model 1, constructed only with the presence of PH, demonstrated statistically significant association between PH and mortality (hazard risk of 2.438; 95% confidence interval of 1.128 – 5.266; p = 0.023). The model 2, performed with presence of PH, age, sex, body mass index, presence of smoking, diabetes mellitus and/or dyslipidemia, and systolic blood pressure, revealed a statistically significant association between both PH and mortality (hazard risk of 3.008; 95% confidence interval of 1.285 – 7.043; p = 0.011) and age and mortality (hazard risk of 1.034 per year of age; 95% confidence interval of 1.000 – 7.068; p = 0.047). The model 3, elaborated with all these covariates and extracellular water, showed a statistically significant association between PH and mortality (hazard risk of 3.164; 95% confidence interval of 1.267 – 7.900; p = 0.014) and a marginally association between age and mortality (hazard risk of 1.034 per year of age; 95% confidence interval of 0.999 – 1.071; p = 0.055). In this last model, any other covariate presented significant association with mortality. When PH was included with albumin, only albumin retained statistical significance (hazard risk of 0.342 per mg/dL; 95% confidence interval of 0.119 – 0.984; p = 0.047) while the presence of PH lost its statistical significance (p = 0.184).

## Discussion

In this cohort of 75 hemodialysis patients, followed up until 10 years, the frequency of PH, evaluated by echocardiography, was 30.6%. As showed in other studies [3,6-9] the presence of PH was associated with a poor prognosis and this fact is accentuated at six years of follow-up. Hemodialysis patients with PH presented differences in cardiac morphology, clinical, and biochemical characteristics when compared with no-PH patients. Cox regression analysis, which included confounding variables, indicated that the effect of PH on mortality is independent of age, sex, body mass index, smoking, diabetes mellitus, dyslipidemia, systolic blood pressure and extracellular water. However, when albumin is included in a model with PH, only albumin retains statistical significance while PH lost its significance as a predictor of mortality. Hence, as lower is the albumin level as higher is the mortality. Therefore, in the current study, the prognostic impact of PH on hemodialysis patients appears to be mediated by lower albumin levels.

The studies that pointed out to the scanty prognosis in hemodialysis patients with PH did not evaluated possible factors, such as albumin or volemic status, that could interfere on mortality in hemodialysis [3,17-21]. Ygla et al [8] showed, by multiple analyses, that besides age, PH was an independent predictor of prognosis, what is in accord with our findings, but their analysis was not adjusted to any biochemical markers of prognosis.

In the present study, PH was associated with markers of volume overload: large left atrium and increased extracellular water. In a multiple logistic regression analysis, only left atrium diameter retained statistically significant association with PH. It is of note that there is a correlation statistically significant between ECF/TBW and left atrium diameter (r = 0.33; p < 0.01) (data not shown). Thus, we hypothesize that volume overload can be important in the physiopathogenesis of PH observed in hemodialysis patients. Corroborating this idea, there are studies that demonstrate higher cardiac output and lower hemoglobin level in PH hemodialysis patients [17-19]. In addition, a recent study by Agarwal [9] showed that left atrial diameter is strongly associated with PH in hemodialysis patients, fact that might reflect chronic volume overload. It is noteworthy that our results also suggest that if right cardiac catheterization had been carried out it possibly would reveal a post-capillary PH which is known as an important risk factor for poor outcome of patients with left heart disease [22].

Although some authors have demonstrated that site and type of vascular access have influence on pulmonary pressure [17-19], we did not find differences regarding to this matter. Also, the association of mineral bone renal disease markers with PH was not observed in the current study differing from Havlucu et al [17] who found increased of parathyroid hormone levels in patients with elevated systolic pulmonary arterial pressure. On the contrary, in accordance with other authors [9,20,21] our PH and no-PH groups were homogeneous concerning calcium, phosphorus, and parathyroid hormone levels.

The tendency to lower albumin level in PH group could be explained by two mechanisms: hemodilution or inflammation. PH group presented markers of overload volume, such as higher extracellular volume, evaluated by bioimpedance, and larger left atrium, while the C-reactive protein was similar in both groups. As C-reactive protein levels were not different between groups, we can suppose that albumin was not decreased by microinflammation; consequently, dilution is the strongest hypothesis to explain the lower albumin levels in PH group. Besides, we stress a statistically significant correlation between ECF/TBW and albumin (r = 0.41 p <0.01) (data not shown). Therefore, these findings point out to hemodilution as the presumable mechanism to explain the propensity to lower albumin level in PH group. Thus, we speculate that volume overload participates in the genesis of increased pulmonary arterial pressure in hemodialysis patients. This physiopathological mechanism of PH might be a peculiarity of chronic kidney disease.

Some limitations must be addressed in the present study. Firstly, it is the small number of patients in our series, but our results encourage its reproduction to confirm these results in other series. Secondly, PH was identified only by echocardiography and not confirmed by right cardiac catheterization. Nevertheless, for ethical reasons, invasive exams for research objectives are not justified. In fact, none of previous studies on PH in hemodialysis patients evaluated pulmonary arterial pressure invasively. Thirdly, the vascular hemodialysis access flux was not measured; however the local and type of access were similar and did not differ statistically between the two groups.

## Conclusion

PH in hemodialysis patients is associated with parameters of volume overload that sheds light on its pathophysiology. Mortality in hemodialysis patients is higher in PH patients, but in a multiple analysis, low level of albumin could explain this association. For the time being, our results suggest that considering the scanty prognostic of hemodialysis patients with PH, strict attention should be taken to avoid volume overload in this population. Perhaps, daily or extended dialysis might help to reach this aim.

## Competing interests

The authors declare that they have no competing interests.

## Authors’ contributions

HHBY, LCM, LST-R, TTQ: conceived and designed the study, interpreted the content analysis, and drafted the manuscript. SGZ-B, BBM: carried out the echocardiography, interpreted the content analysis, and drafted the manuscript. ACK, PB JTC, RJSF: carried out the follow-up, interpreted the content analysis, and drafted the manuscript. All authors read and approved the final manuscript.

## Pre-publication history

The pre-publication history for this paper can be accessed here:

http://www.biomedcentral.com/1471-2369/13/80/prepub
